# Optimal staging system for predicting the prognosis of patients with hepatocellular carcinoma in China: a retrospective study

**DOI:** 10.1186/s12885-016-2420-0

**Published:** 2016-07-07

**Authors:** Lihui Su, Tao Zhou, Zongli Zhang, Xiuguo Zhang, Xuting Zhi, Caixia Li, Qingliang Wang, Chongqi Jia, Wenna Shi, Yanqiu Yue, Yanjing Gao, Baoquan Cheng

**Affiliations:** Department of Gastroenterology, Qilu Hospital, School of Medicine, Shandong University, Jinan, 250012 China; Department of Hepatobiliary Surgery, Qilu Hospital, School of Medicine, Shandong University, Jinan, 250012 China; Department of Intervention, Qilu Hospital, School of Medicine, Shandong University, Jinan, 250012 China; Department of Epidemiology and Health Statistics, Shandong University, Jinan, 250012 China

**Keywords:** Hepatocellular carcinoma, Prognosis, Staging system, Independent predictors, Overall survival

## Abstract

**Background:**

Several staging systems have been developed to evaluate patients with hepatocellular carcinoma (HCC), including the China Staging System (CS), the American Joint Committee on Cancer (AJCC) tumor-node-metastasis (TNM) staging system, and seventh edition; the Barcelona Clinic Liver Cancer (BCLC) staging system, and Cancer of the Liver Italian Program (CLIP) staging system. The optimal staging system for to evaluate patients in China with HCC has not been determined. This study was designed to determine the optimal staging system for predicting patient prognosis by comparing the performances of these four staging systems in a cohort of Chinese patients with HCC.

**Methods:**

This study enrolled 307 consecutive Chinese patients with HCC in Shandong Province. The performances of the CS, TNM, BCLC, and CLIP staging systems were compared and ranked using a concordance index. Predictors of survival were identified using univariate and multivariate Cox model analyses.

**Results:**

The mean overall survival of the patient cohort was 12.08 ± 11.87 months. Independent predictors of survival included tumor size, number of lesions, tumor thromboses, cirrhosis, serum albumin level and serum total bilirubin level. Compared with the other three staging systems, the CS staging system showed optimal performance as an independent predictor of patient survival. The BCLC staging system showed the poorest performance; its treatment algorithm was not suitable for patients in this study.

**Conclusions:**

CS was the most suitable staging system for predicting survival of patients with HCC in China.

**Electronic supplementary material:**

The online version of this article (doi:10.1186/s12885-016-2420-0) contains supplementary material, which is available to authorized users.

## Background

Hepatocellular carcinoma (HCC) is the sixth most common cancer and the third leading cause of cancer deaths worldwide [1]. Approximately 55 % of patients with HCC live in China and the 5-year overall survival (OS) rate is only 7 % [2]. Unlike other solid tumors, the prognosis and treatment options for patients with HCC depend not only on the tumor stage but also on residual liver function [3]. Many staging systems that include both the liver cancer and residual liver function have been developed, including the Cancer of the Liver Italian Program (CLIP); the Barcelona Clinic Liver Cancer (BCLC), the American Joint Committee on Cancer (AJCC) tumor-node-metastasis (TNM), seventh edition and the China Staging (CS) systems [4–8].

Many clinical trials in western countries have evaluated the staging, natural history and prognosis of patients with HCC, with highly variable [9, 10]. Despite China having a greater disease burden than the rest of the world, few studies have been performed in China. Shandong Province, located in the east of China, has a high incidence of HCC. To date, the tumor staging system optimal for evaluating patients with HCC in Shandong province has not been determined. This retrospective study compared the performances of four staging systems, the CLIP, BCLC, AJCC TNM 7th edition, and CS staging systems, in patients with HCC in Shandong Province, China, who were treated at Qilu Hospital of Shandong University. This study also attempted to identify factors independently prognostic of survival in these patients.

## Methods

This study was approved by the institutional ethical committee at Qilu Hospital of Shandong University. All patients or their family provided written informed consent for their clinical records to be stored in the hospital database and used for research.

### Patients

**Fig. 1:**
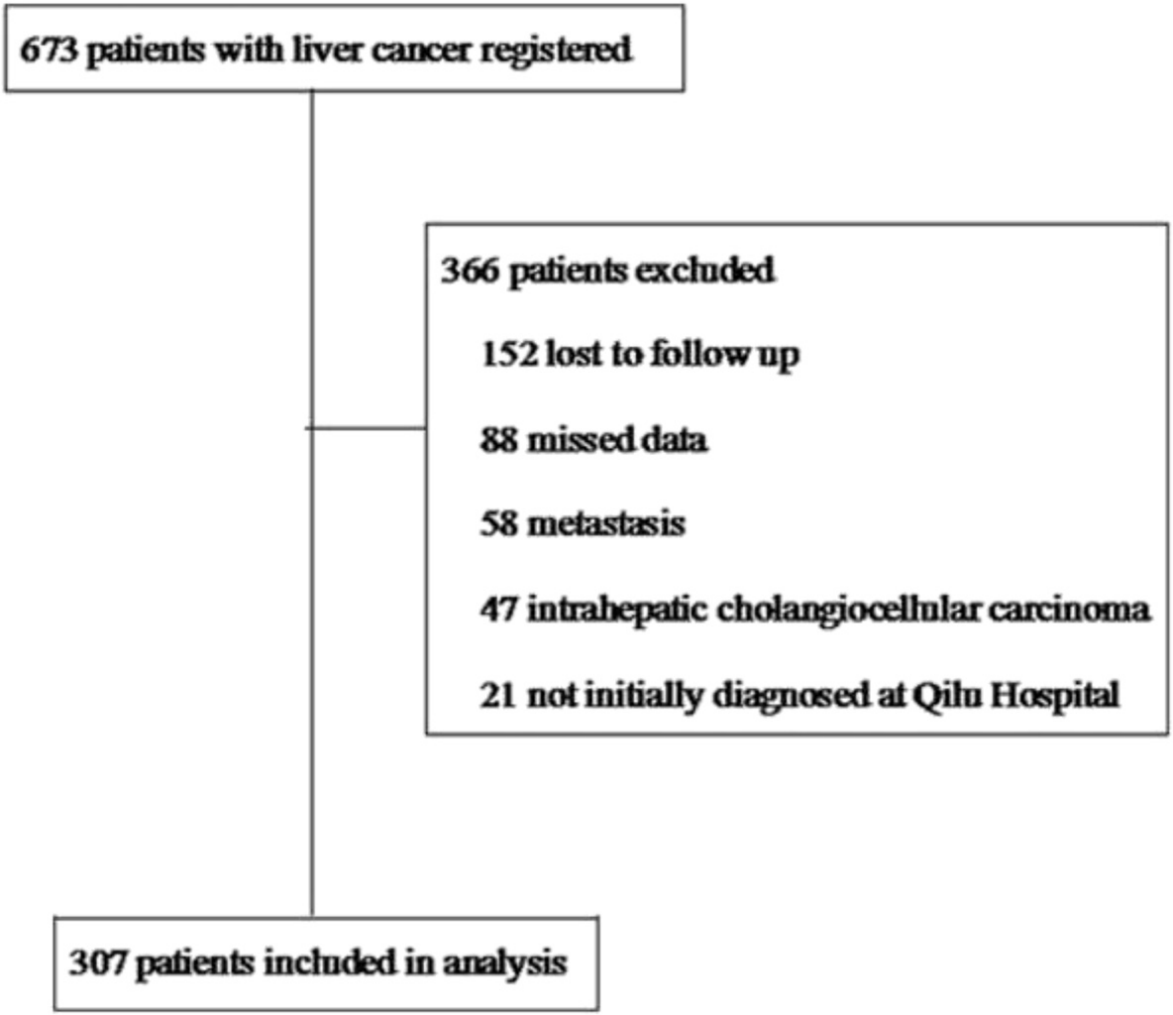
Flow chart of the study

Between January 1, 2010, and October 31, 2014, 673 consecutive patients diagnosed with liver cancer were seen at Qilu Hospital of Shandong University. Of these, 366 patients were excluded, including 152 lost to follow-up, 88 with missing data, 58 with cancer of other organs or tissues metastasis to the liver, 47 diagnosed with intrahepatic cholangiocellular carcinoma, and 21 diagnosed at other centers and referred to Qilu Hospital. The remaining 307 patients with HCC were consecutively enrolled and retrospectively analyzed (Fig. 1).

Baseline information, including the results of clinical examinations, laboratory evaluations, imaging modalities (e.g. computed tomography [CT], magnetic resonance imaging [MRI] and/or ultrasonography), was collected at the time of diagnosis. OS was defined the time from the date of initial diagnosis of HCC to the date of death, last follow-up or the date of censoring (January 1, 2015), whichever came first.

HCC diagnosis was confirmed by histopathological examination of surgical samples or cytologic evaluation of needle biopsy samples (especially if mass less than 2 cm). Alternatively, a diagnosis of HCC was based on the radiologic criteria of the European Association for the Study of the Liver (EASL) [11, 12]. Based on collected data, all included patients were restaged retrospectively according to the CLIP, BCLC, AJCC TNM seventh edition, and CS staging systems.

### Statistical methods

All patients were followed up until death or January 1, 2015. Continuous variables were expressed as mean ± standard deviation (SD), and categorical as frequencies and percentage. Survival outcomes were estimated by the Kaplan–Meier method and compared by the log-rank test.

Staging systems were ranked using the concordance index (c-index), which measures the capacity of the different staging systems to stratify patients with different outcomes: the higher the c-index, the more informative the model was about patient outcomes.

Independent prognostic factors were identified through backward stepwise selection in a Cox regression model. Variables significant (*p* < 0.05) on univariate analysis were included in the multivariate Cox proportional hazards model. Adjusted hazard ratios (HRs) and 95 % confidence intervals (95 % CIs) were calculated.

All statistical analyses were performed using STATA/SE version 13.1 software (Stata Corporation, College Station, TX, USA). All *p*-values were two-sided, and those less than 0.05 were considered statistically significant.

## Results

### Patient characteristics

Of the 307 patients with HCC included in the study, 252 (82.1 %) were male and 55 (17.9 %) were female, with a mean age of 55.43 ± 10.69 years. Among the 307 patients with HCC, 143 (46.6 %) were confirmed by histopathological examination of surgical samples or cytologic evaluation of needle biopsy samples, that the main tumor differentiation was intermediate (40.6 %), and 164 (53.4 %) were diagnosed by the imaging criteria. The most frequent etiology of underlying liver disease was hepatitis B virus (HBV) infection (77.8 %), followed by other etiology (17.6 %), hepatitis C virus (HCV) infection (2.3 %) and alcoholism (1.6 %); only 0.7 % of patients were infected with both HBV and HCV. In total, 65.2 % of patients had a single tumor, with a mean tumor size of 6.18 ± 4.04 cm; and 77.9 % of patients had underlying Child-Pugh class A liver function. Regarding treatment modalities, 56.1 % of patients underwent curative procedures (LR and RFA), whereas TACE, MWA, systemic treatment and supportive care were administrated to 18.9 %, 3.9 %, 4.2 %, 16.9 % of patients, respectively. Of the 307 patients, 120 (39.1 %) had died by the time of the final analysis (January 1, 2015). The mean OS was 12.08 ± 11.87 months (Table 1; Additional files 1 and 2).

**Table 1:** Baseline demographic and clinical characteristics of the 307 patients with hepatocellular carcinoma (Additional files 1 and 2)

Characteristic	
Age, years	
Mean ± SD	55.43 ± 10.69
Range	11–84
Sex, %	
Male	252 (82.1)
Female	55 (17.9)
ECOG PS, %	
0	100 (32.6)
1	146 (47.6)
2	44 (14.3)
3	16 (5.2)
4	1 (0.3)
Etiology, %	
HBV	239 (77.8)
HCV	7 (2.3)
HBV + HCV	2 (0.7)
Alcoholism	5 (1.6)
Other	54 (17.6)
Child-Pugh Grade, %	
A	239 (77.9)
B	56 (18.2)
C	12 (3.9)
Child-Pugh Score, %	
5	176 (57.4)
6	63 (20.5)
7	36 (11.7)
8	9 (2.9)
9	11 (3.6)
10	9 (2.9)
11	3 (1.0)
Hepatic encephalopathy, %	
No	307 (100.0)
Yes	0 (0)
Ascites, %	
No	219 (71.4)
Little	56 (18.2)
Middle	24 (7.8)
Large	8 (2.6)
Portal hypertension, %	
No	245 (79.8)
Yes	62 (20.2)
Cirrhosis, %	
No	97 (31.6)
Yes	210 (68.4)
Laboratory values, mean ± SD	
Total bilirubin (μmol/l)	24.55 ± 50.93
Albumin (g/l)	38.53 ± 5.78
Prothrombin time (sec)	0.19 ± 0.80
AFP (ng/ml), %	
< 400	211 (68.7)
≥ 400	96 (31.3)
Tumor characteristics	
Tumor size (mean ± SD, cm)	6.18 ± 4.04
Number of lesions, %	
1	200 (65.2)
2–3	29 (9.4)
≥ 4	78 (25.4)
Lobar involvement, %	
Unilobar	164 (53.4)
≥ bilobar	143 (46.6)
Tumor morphology, %	
Uninodular	147 (47.9)
Multinodular	77 (25.1)
Massive, diffuse	83 (27.0)
Vascular and/or organ invasion, %	
No	235 (76.6)
Portal/hepatic vein	52 (16.9)
Other vascular	9 (2.9)
Organ invasion	11 (3.6)
N, %	
No	252 (82.1)
Yes	55 (17.9)
M, %	
No	278 (90.6)
Yes	29 (9.4)
Tumor thrombosis, %	
None	247 (80.4)
Portal stem vein	26 (8.5)
Inferior vena cava	6 (1.9)
Hepatic vein branches	2 (0.7)
Portal vein branches	16 (5.2)
Vessel	3 (1.0)
Hepatic duct	2 (0.7)
Inferior vena cava branches and Portal vein branches and/or Hepatic vein branches	5 (1.6)
Current outcomes, %	
Dead	120 (39.1)
Alive	187 (60.9)
OS, mean ± SD, months	12.08 ± 11.87

Patients were classified into stage groups according to the four staging systems. According to the BCLC staging system, 63.8 % of referred patients had advanced stage tumor stages. In contrast, the different AJCC TNM stages were more evenly distributed, and 96.7 % of patients had CLIP scores ≤ 4. According to the CS staging system, 29.7, 37.1, and 33.2 % of patients had stages I, II, and III disease, respectively (Table 2).

**Table 2:** Tumor staging information of the 307 patients with hepatocellular carcinoma

Staging system	Patients (%)
CLIP	
0	95 (30.9)
1	86 (28.0)
2	42 (13.7)
3	53 (17.3)
4	21 (6.8)
5	8 (2.6)
6	2 (0.7)
BCLC	
0	10 (3.3)
A	45 (14.7)
B	32 (10.4)
C	196 (63.8)
D	24 (7.8)
TNM^a^	
I	133 (43.3)
II	41 (13.4)
IIIA	15 (4.9)
IIIB	37 (12.0)
IIIC	11 (3.6)
IVA	41 (13.4)
IVB	29 (9.4)
CS	
Ia	49 (16.0)
Ib	42 (13.7)
IIa	51 (16.6)
IIb	63 (20.5)
IIIa	90 (29.3)
IIIb	12 (3.9)

^a^Seventh edition

### Baseline predictors of survival

Univariate analysis showed that Child-Pugh grade, tumor size and number, serum total bilirubin and AFP concentrations, tumor thromboses, and cirrhosis were significantly associated with OS (Table 3). Multivariate analysis found that tumor size, number of lesions; serum total bilirubin level and tumor thromboses were the most accurate independent predictors of OS (*p* ≤ 0.001 each). In addition, cirrhosis and albumin were also predictive of reduced OS (Table 4).

**Table 3:** Univariate analyses of factors independently prognostic of overall survival in the 307 patients with hepatocellular carcinoma

Variable	Coefficient	SE	P	HR	95 % CI
Sex	0.39	0.28	0.156	1.48	0.86–2.54
Age	−0.09	0.08	0.293	0.92	0.78–1.08
ECOG PS	0.74	0.10	0.000	2.10	1.74–2.54
Tumor size	0.15	0.02	0.000	1.16	1.12–1.21
Number of lesions	0.58	0.10	0.000	1.78	1.47–2.17
Lobar involvement	1.16	0.20	0.000	3.20	2.18–4.70
Tumor formation	0.70	0.11	0.000	2.01	1.63–2.49
Ascites	0.44	0.10	0.000	1.55	1.27–1.89
Total bilirubin	0.68	0.14	0.000	1.98	1.51–2.60
Albumin	−0.10	0.02	0.000	0.91	0.88–0.94
Child-Pugh Grade	0.86	0.16	0.000	2.37	1.72–3.25
alpha-fetoprotein	0.72	0.18	0.000	2.05	1.43–2.94
hepatitis B virus	−0.37	0.21	0.081	0.69	0.46–1.05
hepatitis C virus	−0.55	0.59	0.347	0.58	0.18–1.82
Alcoholism	0.53	0.59	0.366	1.70	0.54–5.36
Other	0.42	0.23	0.066	1.53	0.97–2.39
Lymph node metastasis	0.81	0.21	0.000	2.26	1.48–3.43
Distant metastasis	1.37	0.22	0.000	3.93	2.56–6.05
Tumor thromboses	1.31	0.20	0.000	3.71	2.50–5.50
Portal hypertension	0.30	0.22	0.173	1.35	0.88–2.09
Cirrhosis	−0.80	0.18	0.000	0.45	0.31–0.64
Vascular/organ invasion					
Portal/hepatic vein	1.31	0.21	0.000	3.72	2.47–5.60
Others	0.79	0.33	0.015	2.21	1.17–4.18

**Table 4:** Multivariate analysis of factors prognostic of overall survival in the 307 patients with hepatocellular carcinoma

Variables	Coefficient	SE	P	HR	95 % CI
Cirrhosis	−0.56	0.21	0.008	0.57	0.38–0.87
Tumor size	0.11	0.02	0.000	1.12	1.07–1.17
Number of lesions	0.36	0.11	0.001	1.44	1.16–1.79
Total bilirubin	0.56	0.16	0.000	1.74	1.28–2.37
Albumin	−0.04	0.02	0.019	0.96	0.93–0.99
Tumor thromboses	0.70	0.22	0.001	2.01	1.31–3.09

### Survival comparisons among staging groups

**Fig. 2:**
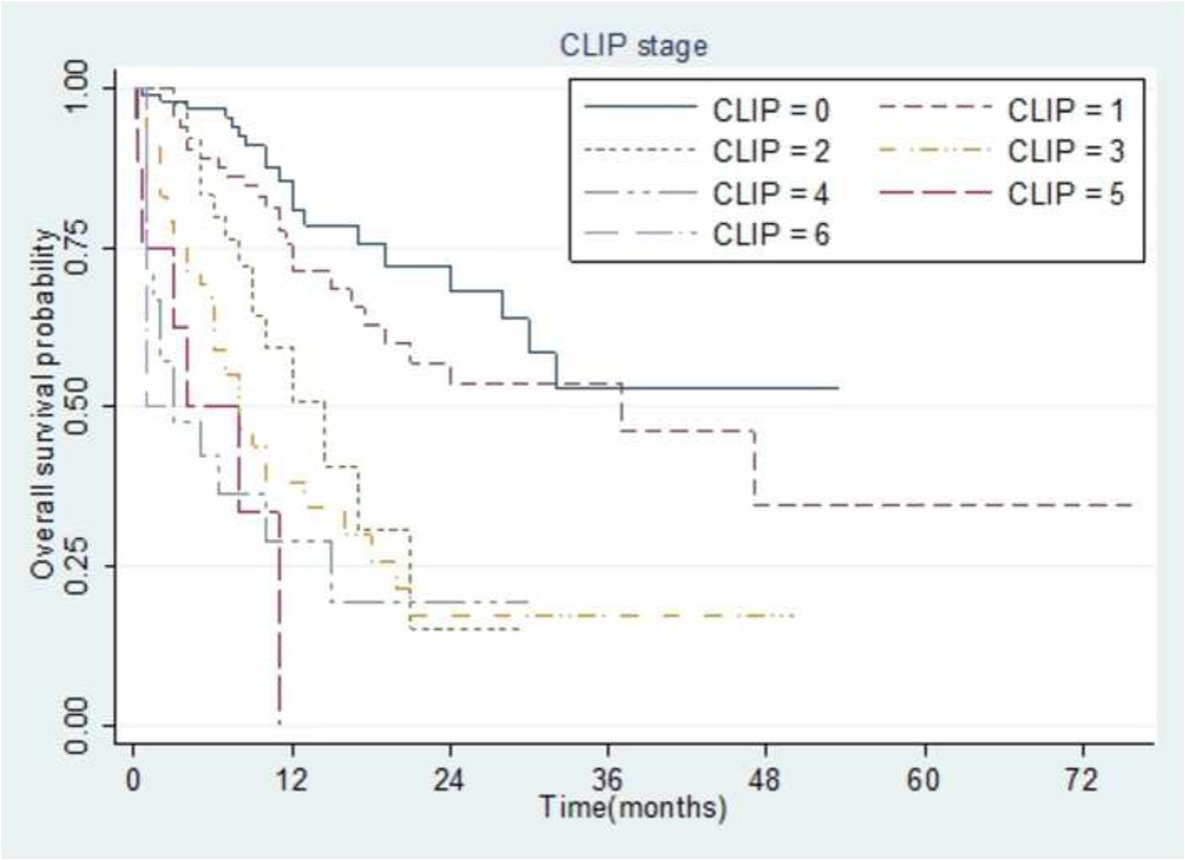
Kaplan–Meier analysis of overall survival in 307 patients with hepatocellular carcinoma stratified according to the Cancer of the Liver Italian Program (CLIP) staging system. All differences between groups wee statistically significant (*p* < 0.001)

**Fig. 3:**
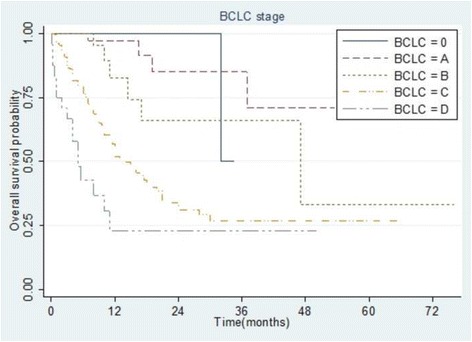
Kaplan–Meier analysis of overall survival in 307 patients with hepatocellular carcinoma stratified according to the Barcelona Clinic Liver Cancer staging system. All differences between groups were statistically significant (*p* < 0.001)

**Fig. 4:**
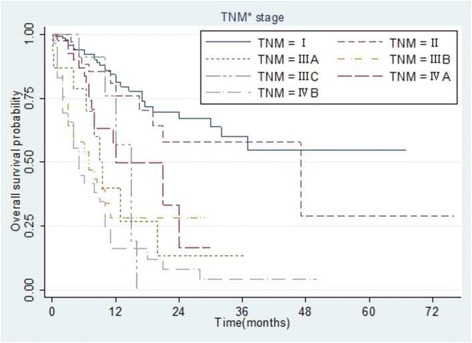
Kaplan–Meier analysis of overall survival in 307 patients with hepatocellular carcinoma stratified according to the AJCC TNM seventh edition staging system. All differences between groups were statistically significant (*p* < 0.001)

**Fig. 5:**
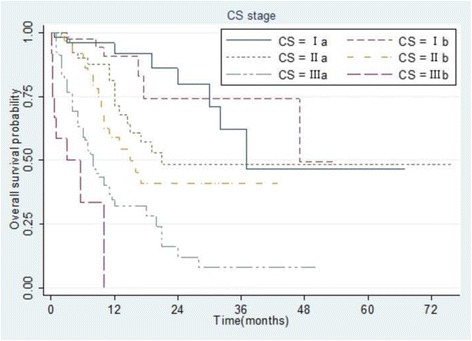
Kaplan–Meier analysis of overall survival in 307 patients with hepatocellular carcinoma stratified according to the Chinese staging system. All differences between groups were statistically significant (*p* < 0.001)

Survival curves were generated by Kaplan–Meier method for each of the four staging systems. Stage groupings of all four staging systems were significantly predictive of OS (*p* < 0.001 each), although some overlapping of survival curves was observed (Figs. 2, 3, 4 and 5).

### Ranking of discriminatory ability of staging system

The prognostic ability of the different staging systems was compared by calculating the c-index of each. The CS staging system had the highest c-index (0.75; 95 % CI, 0.71–0.80), followed by CLIP (0.74; 95%CI, 0.69–0.79), the AJCC TNM seventh edition (0.70; 95 % CI, 0.65–0.75), and BCLC (0.69; 95 % CI, 0.65–0.73) staging systems. There was a significant difference between prognostic ability of the CS staging system compared with BCLC staging system (*p* = 0.031). However, it was no statistically difference among the others (CS compared with CLIP, *p* = 0.130; CS compared with the AJCC TNM seventh edition, *p* = 0.746; CLIP compared with the AJCC TNM seventh edition, *p* = 0.243; CLIP compared with BCLC, *p* = 0.661; the AJCC TNM seventh edition compared with BCLC, *p* = 0.080) (Table 5).

**Table 5:** Ranking of staging systems by concordance indices in patients with hepatocellular carcinoma

Rank	System	C-index	95 % CI
1	CS	0.75	0.71–0.80
2	CLIP	0.74	0.69–0.79
3	TNM^a^	0.70	0.65–0.75
4	BCLC	0.69	0.65–0.73

^a^Seventh edition

## Discussion

The predominant etiology of HCC in patients in Shandong Province China was HBV infection. This study of factors independently prognostic of OS in this population found that tumor extent (e.g. tumor size, number of liver lesions, and tumor thromboses), hepatic function (serum total bilirubin concentration and serum albumin level), cirrhosis were independent baseline predictors of OS. Of this patient population, 68.4 % had underlying cirrhosis, which was strongly associated with OS, and 70–80 % showed histological evidence of liver cirrhosis. AFP was again of limited use in this study, because it was proven to be both not sensitive enough to identify early stage HCC and not specific enough to avoid unnecessary recall procedures, so AFP test has been dropped from the latest Western guideline for the clinical diagnosis of HCC [11–13]. We also founded that serum total bilirubin concentration, serum albumin level and greater tumor extent were related to poor prognosis variables, indicating that the long-term survival of patients with HCC was associated not only with the tumor but with liver function [3, 14–16].

CS was a new staging system proposed by Chinese Society of Liver Cancer (CSLC) for the patients with hepatocellular carcinoma and was initially launched in 2001. The CS staging system combined hepatic function, as defined by Child-Pugh classification, and tumor extent, as defined by adjusted TNM stage, that the parameters included tumor size and tumor location, thrombosis (portal vein, inferior vena cava and biliary duct), lymph node metastasis, distant metastasis and the Child-Pugh classification [8]. It classified stages of disease into six subgroups, from Ia to IIIb (Table 6). This study found that the CS staging system had the highest c-index and there was a significant difference between prognostic ability of the CS staging system compared with BCLC staging system (*p* = 0.031). So, the CS staging system was optimal in distinguishing survival categories in patients with HCC in Shandong Province, China. The CS staging system was the most prognostic in our cohort because it included the independent predictors of survival we had identified. These included serum concentration of total bilirubin and serum albumin level, parameters of Child-Pugh grade, which can reflect the residual hepatic function of the patients with HCC; and tumor stage (tumor size, portal vein thromboses, and number of liver lesions). In contrast, the BCLC staging system showed the poorest performance, despite its having been viewed as the standard classification that is used for trial design and clinical management of patients with HCC [17]. Several reasons may explain the unsuitability of the BCLC staging system for Chinese patients with HCC. First, studies have shown that the performance of the BCLC staging system may be better in patients with early than late stage disease [18, 19]. However, 63.8 % of the patients in our study had advanced stage disease (BCLC stage C), limiting the discriminatory ability of BCLC staging. Second, the natural history of HCC may vary by underlying etiology. The primary cause of HCC in western countries is HCV infection, whereas the primary cause of HCC in our population was HBV infection (77.8 %). Therefore, the ability of BCLC staging to stratify Asian patients with HBV-associated HCC remains unclear [19, 20].

**Table 6:** The classification criteria of China staging system

Stage	Tumor size (cm) and location	Thrombosis	N	M	Child-Pugh score
Ia	single, ≤ 3	absent	absent	absent	A
Ib	unilobar, ≤ 5	absent	absent	absent	A
IIa	unilobar, ≤ 10; or bilobar, ≤ 5	absent	absent	absent	A
IIb	unilobar, >10; or bilobar, > 5; any	absent; portal vein, or inferior vena cava, or biliary duct branches	absent	absent	
			absent	absent	A or B
IIIa	any;	portal vein, or inferior vena cava, or biliary duct stem;	any	any	A or B
	any;	any;	present	any	
	any	any	any	present	
IIIb	any	any	any	any	C

*N* lymph node metastasis, *M* distant metastasis

This study had several potential limitations. First, it was retrospective in design. Moreover, 152 patients were lost to follow up and data were missing for 88. However, many Chinese people live in the countryside, making communication difficult. Thus, there may have been potential bias in patient selection. Secondly, this was a single-center study involving patients admitted consecutively to Qilu Hospital of Shandong University for treatment. However, our study had several strengths. Complete data were obtained from a large number of patients. Moreover, the follow-up period was relatively long, and the epidemiological characteristics of our cohort were consistent with those reported in other studies of Chinese patients with HCC [20, 21].

## Conclusions

Of the four HCC staging systems evaluated, the CS staging system was the most informative in predicting survival for patients with HCC in Shandong Province. The poor performance of the BCLC staging system in this cohort suggests its unsuitability for evaluating Chinese patients with HCC. We also found that tumor size, number of lesions, tumor thromboses, serum total bilirubin level; albumin and cirrhosis were the accurate independent predictors of OS.

## Abbreviations

95 % CI, 95 % confidence interval; AFP, alpha-fetoprotein; AJCC, American Joint Committee on Cancer; BCLC, Barcelona Clinic Liver Cancer; c-index, concordance index; CLIP, Cancer of the Liver Italian Program; CS, China Staging System; CSLC, Chinese Society of Liver Cancer; CT, computed tomography; ECOG PS, Eastern Cooperative Oncology Group performance status; HBV, hepatitis B virus; HCC, hepatocellular carcinoma; HCV, hepatitis C virus; HR, hazard ratio; M, Distant metastasis; MRI, magnetic resonance imaging; N, Lymph node metastasis; OS, overall survival; SD, standard deviation; SE, standard error; TNM, tumor-node-metastasis

## Additional files

Additional file 1: Availability of data and material(1). (XLS 99 kb) Additional file 2: Availability of data and material(2). (DOC 30 kb)
