# Supplementary material for: Optimal staging system for predicting the prognosis of patients with hepatocellular carcinoma in China: a retrospective study
Source: BMC Cancer. 2016 Jul 7;16:424. doi: 10.1186/s12885-016-2420-0 (PMC4937540; doi:10.1186/s12885-016-2420-0)
Supplement: Additional file 2: — Availability of data and material(2). (DOC 30 kb) [file 12885_2016_2420_MOESM2_ESM.doc]

**The statement of original data**

**1. Patients 1-307**

**2. Sex women: 1; man: 2**

**3. Age 11-84 years old**

**4. ECOG PS 0-4**

**5. Tumor size** **(cm) no: -4**

**6. Number of lesions one: 1**

**Two-three: 2**

**≥ four: 3**

**7. Lobar involvement Unilobar: 1**

**≥ bilobar:** **2**

**8. Tumor morphology Uninodular: 1**

**Multinodular: 2**

**Massive, diffuse:** **3**

**9. Vascular and/or organ invasion No: 0**

**Portal/hepatic vein: 1**

**Other vascular: 2**

**Organ invasion: 3**

**10. Tumor differentiation Low: 1**

**Intermediate：2**

**High: 3**

**Intermediate-low: 4**

**High-intermediate: 5**

**No: -6**

**11. Hepatic encephalopathy No: 0**

**12. Ascites No: 0**

**Little: 1**

**Middle: 2**

**Large: 3**

**13. Total bilirubin (µmol/l)**

**14. Albumin (g/l)**

**15. Prothrombin time (sec)**

**16. Child-Pugh Grade A: 1**

**B: 2**

**C: 3**

1. **Child-Pugh Score 5-11**

**18. AFP (ng/ml) No: -1**

**19. HBsAg (-): 0; (+): 1**

**20. HCVAb (-): 0; (+): 1**

**21. Alcohol (-): 0; (+): 1**

**22. Other (-): 0; (+): 1**

**23. Lymph node metastasis No: 0; Yes: 1**

**24. Distant metastasis No: 0; Yes: 1**

**25. Tumor thrombosis No: 0**

**Portal stem vein: 1**

**Inferior vena cava: 2**

**Common bile duct: 3**

**Hepatic vein branches: 4**

**Portal vein branches: 5**

**Vessel: 6**

**Hepatic duct: 7**

**Inferior vena cava branches**

**and Portal vein branches**

**and/or Hepatic vein branches: 8**

**26. Portal hypertension No: 0; Yes: 1**

**27. Cirrhosis No: 0; Yes: 1**

**28. Overall survival(months)**

**29. CLIP No: -2**

**0-6**

**30. BCLC 0 stage: 0**

**A stage: 1**

**B stage: 2**

**C stage: 3**

**D stage: 4**

**31. TNM Ⅰ stage: 1**

**Ⅱ stage: 2**

**ⅢA stage: 3**

**ⅢB stage: 4**

**ⅢC stage: 5**

**ⅣA stage: 6**

**ⅣB stage: 7**

**32. CS Ⅰa stage: 1**

**Ⅰb stage: 2**

**Ⅱa stage: 3**

**Ⅱb stage: 4**

**Ⅲa stage: 5**

**Ⅲb stage: 6**

**33.Current outcomes Alive: 1; Dead: 0**

**34. Treatment modality Liver resection: 1**

**TACE：2**

**RFA：3**

**MWA：4**

**Systematic treatment:5**

**Support care：6**
